# A study of wrist-worn activity measurement as a potential real-world biomarker for late-life depression

**DOI:** 10.1017/S0033291716002166

**Published:** 2016-09-26

**Authors:** J. T. O'Brien, P. Gallagher, D. Stow, N. Hammerla, T. Ploetz, M. Firbank, C. Ladha, K. Ladha, D. Jackson, R. McNaney, I. N. Ferrier, P. Olivier

**Affiliations:** 1 Department of Psychiatry, University of Cambridge, Cambridge, UK; 2 Institute of Neuroscience, Newcastle University, Newcastle upon Tyne, UK; 3 Newcastle University Institute for Ageing, Newcastle upon Tyne, UK; 4 Open Lab, Newcastle University, Newcastle upon Tyne, UK

**Keywords:** Activity, ageing, depression, magnetic resonance imaging, neuropsychology

## Abstract

**Background:**

Late-life depression (LLD) is associated with a decline in physical activity. Typically this is assessed by self-report questionnaires and, more recently, with actigraphy. We sought to explore the utility of a bespoke activity monitor to characterize activity profiles in LLD more precisely.

**Method:**

The activity monitor was worn for 7 days by 29 adults with LLD and 30 healthy controls. Subjects underwent neuropsychological assessment and quality of life (QoL) (36-item Short-Form Health Survey) and activities of daily living (ADL) scales (Instrumental Activities of Daily Living Scale) were administered.

**Results:**

Physical activity was significantly reduced in LLD compared with controls (*t* = 3.63, *p* < 0.001), primarily in the morning. LLD subjects showed slower fine motor movements (*t* = 3.49, *p* < 0.001). In LLD patients, activity reductions were related to reduced ADL (*r* = 0.61, *p* < 0.001), lower QoL (*r* = 0.65, *p* < 0.001), associative learning (*r* = 0.40, *p* = 0.036), and higher Montgomery–Åsberg Depression Rating Scale score (*r* = −0.37, *p* < 0.05).

**Conclusions:**

Patients with LLD had a significant reduction in general physical activity compared with healthy controls. Assessment of specific activity parameters further revealed the correlates of impairments associated with LLD. Our study suggests that novel wearable technology has the potential to provide an objective way of monitoring real-world function.

## Introduction

There is an important, bidirectional relationship between physical activity and late-life depression (LLD), with evidence that LLD is associated with impairments in activities of daily living (ADL) and a reduction in gross physical activity. It has also been consistently demonstrated that LLD is associated with psychomotor slowing, widespread cognitive impairments (that can persist despite clinical recovery), reduced quality of life (QoL) and structural brain changes, including an increase in white matter lesions (Colloby *et al.* 2011). However, the extent to which changes in ADL and QoL, imaging abnormalities and cognitive impairments demonstrated in clinical/laboratory settings relate to depression and physical activity in LLD in the real world remains unclear. The major barrier to elucidating the nature of this relationship has been devising an acceptable and validated way of accurately measuring activities in the real world on an ongoing basis, especially in those with depression. While activity levels have been measured using wrist- and chest-worn actigraphs in younger depressed subjects (Burton *et al.* 2013), few previous studies have specifically examined the utility of these devices to the assessment of community-dwelling older depressed subjects. Furthermore, while the use of actigraphy to assess day-to-day physical activity has great potential, there are important limitations to the interpretability of the data they produce due to a lack of agreement as what constitutes light/moderate/intense activity in older adults and the likelihood that these arbitrary categorizations reduce data fidelity (van Hees, 2012; Kim *et al.* 2013*a*). Through the rapid development of this technology, it has been suggested that far more insightful comparisons can be drawn by utilizing analysis of raw output from accelerometers (i.e. direct sensor readings), rather than the categorical data derived from actigraphy (i.e. aggregated epoch values such as step counts etc.; Sabia *et al.* 2014). Therefore, in this study we sought to develop and collect feasibility data from a novel wrist-worn device that, unlike an actigraph, allows the analysis of direct sensor readings. Using these readings, we sought to characterize fine-detail activity levels in depressed subjects and healthy comparison subjects. Our primary hypothesis was that gross measures of physical activity would be reduced in LLD patients compared with healthy controls. We further hypothesized that analysis of raw accelerometer data would demonstrate altered movement signatures in LLD patients. Based on analysis of raw accelerometer data, our exploratory hypotheses were that 24 h patterns of fine-grained physical activity in the real world would correlate with measures of social function, ADL, QoL, neuropsychological function, severity of depression and magnetic resonance (MR)-derived measures of regional brain volumes.

## Method

### Participants

A total of 29 participants aged over 60 years and who fulfilled Diagnostic and Statistical Manual of Mental Disorders, 4th edition (DSM-IV) criteria for current major depression, as assessed using the Mini-International Neuropsychiatric Interview (M.I.N.I.; Sheehan *et al.* 1998), were recruited from secondary care services covering four geographically based secondary catchment areas across the North East of England. A comparison group (*n* = 30) of similarly aged controls with no self-reported history of depression or current depression as measured using the M.I.N.I were recruited. These came predominantly from a volunteer database maintained by the North East England Clinical Research Network. Individuals with evidence of a severe or unstable physical illness (e.g. recent significant cardiac events, insulin-dependent diabetes mellitus, untreated hypothyroidism, cancer), known cognitive impairment or dementia, Mini Mental State Examination (MMSE; Folstein *et al.* 1975) score <24, acquired brain injury or stroke were excluded from the study. Other exclusion criteria were: recent history or current evidence of substance abuse (e.g. alcohol, drugs); uncorrected visual or auditory sensory deficits; and history of electroconvulsive therapy (ECT) in the past 6 months (clinical sample) or any history of ECT (control sample). All participants had English as a first language. The National Research Ethics Service committee for the North East of England approved the study. All patients and controls gave written informed consent to participate after the study protocol had been fully explained.

### Materials

**Fig. 1.**
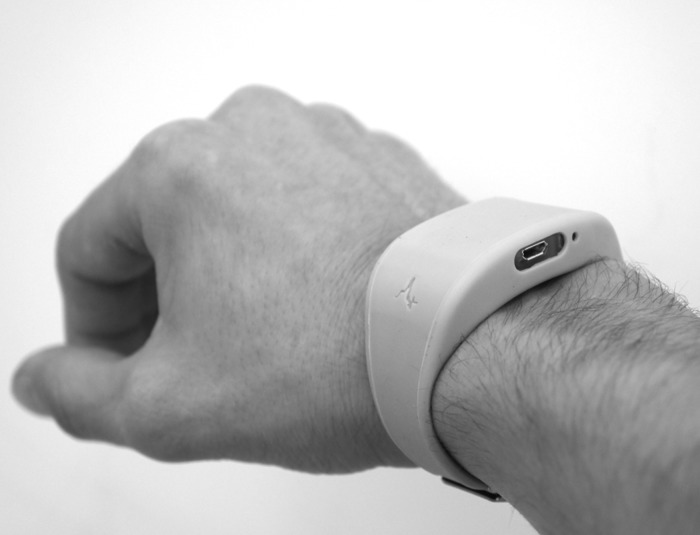
The wearable monitor.

The wearable monitor was a stand-alone, wrist-mounted device that recorded physical activity via three accelerometers. The device was designed to be as unobtrusive as possible to minimize non-adherence and to allow the capture of raw data from a naturalistic environment (Fig. 1). The study device†^1^ included a tri-axial accelerometer, internal data storage and a lithium ion battery lasting for a mean duration of 5 days. These instruments, their controllers and the accompanying printed circuit board were encased in a thermoplastic housing, which was then injected with a resin compound to ensure water resistance. The device itself was held by an adjustable silicone wristband, with a stainless-steel fastening mechanism to allow for comfortable, hypoallergenic, wrist-mounted use.

### Procedure

Consenting eligible participants were screened at a baseline assessment during which demographic information and self-report histories of current medication, physical and mental health were recorded. Assessments included: the M.I.N.I., the Montgomery–Åsberg Depression Rating Scale (MADRS; Montgomery & Åsberg, 1979); the 15-item Geriatric Depression Scale (GDS-15; Sheikh & Yesavage, 1986); the MMSE; the National Adult Reading Test (NART; Nelson, 1982) and the Edinburgh Handedness Inventory (Oldfield, 1971). Participants received three further visits, all of which took place in their homes. On day 1, the study device was delivered. Assessments of mood (MADRS and GDS-15) were repeated along with measures of social functioning and physical/mental wellbeing: Short-Form Health Survey (SF-36; Ware & Sherbourne, 1992) and Instrumental Activities of Daily Living Scale (IADL; Lawton & Brody, 1969). Because the device battery life was less than the 7-day study period, between days 2 and 6 the initial study device was swapped for a second, identical, fully charged device at a home visit. After a full 7 days, the study device was collected and assessments of mood (MADRS and GDS-15) were repeated.

#### Neuropsychological assessment

Subjects underwent a comprehensive neuropsychological test battery during the study period consisting of: a digit span (DS) task, a digit symbol substitution task (DSST), a computer-administered facial emotion processing task (FERT; Adams *et al.* 2015), the two-part Trail Making task (TMA, TMB), the Rey Auditory Verbal Learning task (RAVL), the FAS verbal fluency task (FAS), and four tasks from the Cambridge Neuropsychological Test Automated Battery (CANTAB): paired associates learning (PAL), spatial span (SSP), spatial working memory (SWM), affective go/no-go (AGN). Pen-and-paper tasks were administered according to standardized instructions. CANTAB tasks were carried out according to the protocol manual on a laptop fitted with a 12.5” colour touchscreen. The FERT was carried out on the same laptop using the attached keyboard modified with labels for the target emotions.

#### MR imaging (MRI) scanning protocol

All eligible subjects were offered participation in the MRI substudy. In all, 15 patients and 15 controls underwent a 3-Tesla MRI scan on a Philips Achieva clinical MR system using an eight-channel head coil. This included T1-weighted whole brain magnetization-prepared rapid gradient-echo (MPRAGE) images acquired in the sagittal plane (repetition time = 8.3 ms, echo time = 4.6 ms, flip angle = 8°, inversion delay = 1250 ms, 216 × 208 matrix, slice thickness = 1.0 mm) yielding 180 slices through the brain. A fluid attenuated inversion recovery (FLAIR) sequence (repetition time = 11 000 ms, echo time = 125 ms, inversion recovery = 2800 ms, turbo spin echo factor = 27; refocus angle = 120°, slice thickness = 3 mm) yielded 50 slices through the brain. MR images were processed using SPM8 (http://www.fil.ion.ucl.ac.uk/spm/) and Matlab 2012b (MATLAB6.1; The MathWorks Inc., USA). Hippocampal volumes were measured using an automated segmentation technique (Firbank *et al.* 2008). Volumetric measurements of white matter hyperintensity (WMH) were obtained for each individual using a previously validated method (Firbank *et al.* 2004).

### Data analysis

Data were analysed with SPSS 21 (IBM Corp.; USA) and R (http://www.R-project.org/). Q-q plots were used to establish the normality of the data. Group differences were assessed using independent *t* tests. To militate against the problem of multiple comparisons with the neuropsychological data, composite average *Z* scores were created for five conceptual domains:

Executive working memory: total score from FAS verbal fluency, total score from backward DS test, between errors (4–10 boxes) from CANTAB SWM.

Attention and psychomotor speed: trails A total time, DSST total time.

Short-term memory: total score from forward DS test, span length from CANTAB SSP.

General memory: total from RAVL presentations 1–5, maximum recall from delayed condition of RAVL, total errors (adjusted) from CANTAB PAL, standardized profile score from RMBT.

Emotional processing: median latency of correct responses on the CANTAB AGN, proportion of correct to incorrect answers from the FERT.

Patient data were cantered against control group *Z* scores by subtracting the mean for control participants from the raw score of each depressed patient then dividing the result by the standard deviation of the control group. A grand mean *Z* score was calculated for performance across all tests. Multivariate analysis, covarying for NART intelligence quotient (IQ), was used to assess group differences in domain scores and missing values were imputed with group mean values.

#### Analysis of physical activity data from the study device

##### Pre-processing

Each recording from the study device was subjected to pre-processing, comprising the following steps: (i) autocalibration as described in van Hees *et al.* (2014); (ii) interpolation of the recordings to a frequency of 50 Hz using bi-cubic interpolation; (iii) estimation of the acceleration magnitude; and (iv) band-pass filtering to remove the effect of gravity using a Butterworth filter with cut-off frequencies of 0.2 and 15 Hz, respectively. Finally, changeover days (when participants were assisted in changing from one device to the next) were combined into a single day. Changeover typically lasted below 1 min, resulting in minimal data loss. Wear-time was estimated using the approach described in van Hees *et al.* (2011), where each 30 min segment of recording is classified as ‘wear-time’ if the standard deviation of the acceleration magnitude exceeds a fixed threshold of 13 m***g*** (***g*** = 9.81 m/s^2^).

##### Exclusion criteria for days of recording

A day of recording from a participant qualified for further analysis if the device was worn for more than half the day, if the device calibration was successful, and if malfunctioning could be ruled out (e.g. due to memory corruption). One recording from the control group was excluded from analysis due to calibration failure to leave 29 healthy control recordings. The majority of first and last days of recordings were also excluded as the device was worn for less than half the day. A total of 171 days were included for the depressed group (average 5.9 days per participant), and a total of 210 days were included for the control group (average 7.2 days per participant). We observed high compliance in both the depressed (92.2%) and the control group (92.3%) on those days that were included in the analysis.

##### Measures of physical activity

For each 1 min of recording of a participant we extracted the following characteristics (Godfrey *et al.* 2008): (i) ‘physical activity’ as the mean acceleration magnitude, a measure for how much, on average, the device was accelerated within 1 min; (ii) ‘jerk’ as the mean of the first derivative of acceleration magnitude, estimated using a five-sample linear regression. Jerk reflects the ‘suddenness’ of movements, where ‘quick’ movements lead to a higher jerk or change in acceleration over time; (iii) ‘entropy’ as the statistical entropy over the 1 min of acceleration magnitude. Entropy is a measure for disorder that reflects how ‘structured’ a movement is. Low entropy is associated with stationarity, slow, and repetitive or self-similar movements. High entropy is associated with less predictable, more energetic movements like gesturing.

##### Activity data analysis

Analysis of activity data was carried out using Matlab 2015a (The MathWorks Inc., USA). We constructed an average day for each participant by estimating the mean of each of physical activity, jerk and entropy for the 1440 min in the day across all days of recording. Non-wear time was excluded from the calculation of the means for each minute. Potentially confounding effects of age, body mass index (BMI) and pre-morbid IQ were removed from the estimated measures through a linear regression of each variable with confounding variables. Only the residuals of this regression were retained and added to the overall mean, removing confounding effects in the process. Group differences were assessed using two-tailed independent *t* tests on the mean across the average day. Further exploratory analysis was conducted where the mean night-time activity (00.00–06.00 hours) and mean daytime activity (06.00–24.00 hours) were correlated with key study variables using partial correlation analysis.

## Results

### Subject characteristics

The groups did not differ significantly in age, sex or living status, but there were group differences in pre-morbid IQ, MMSE and BMI (Table 1). Subsequent analysis of neuropsychological data covaried for NART IQ and correlations with physical activity data controlled for age, sex, NART IQ and BMI.

**Table 1.** Demographic characteristics and group comparisons

	LLD patients		Healthy controls		Analysis		
	Mean (s.d.)	*n*	Mean (s.d.)	*n*	Statistics	df	*p*
Demographics							
Age, years	74.0 (6.0)	29	73.9 (5.9)	30	*t* = 0.09	58	0.93
Female		21		22			
Lives alone		18		12	χ^2^ = 2.9	1	0.09
Education, years	10.9 (2.2)	29	13.5 (3.3)	30	*t* = −3.47	57	<0.001
NART IQ score	109.0 (10.2)	29	114.5 (10.2)	30	*t* = −2.1	57	<0.05
MMSE score	27.7 (1.8)	29	28.9 (1.1)	30	*t* = −3.08	57	<0.005
Number of previous episodes	6.5 (13.0)	26					
BMI, kg/m^2^	30.8 (6.8)	28	26.4 (3.9)	30	*t* = 3.05	55	<0.005
Neuropsychological *Z* scores							
Executive working memory	−1.2 (1.8)	29	0 (0.7)	30	*F* = 6.56	1,59	0.01
Attention and psychomotor speed	−2.0 (2.8)	28	0 (0.9)	30	*F* = 11.09	1,59	<0.005
Short-term memory	−0.5 (0.8)	29	0 (0.8)	30	*F* = 2.08	1,59	0.15
General memory	−1.0 (1.1)	29	0 (0.8)	30	*F* = 11.1	1,59	<0.005
Emotional processing	−0.1 (0.8)	28	0 (1.0)	29	*F* = 0.06	1,59	0.81
Grand score	−0.9 (0.9)	29	0 (0.5)	30	*F* = 17.47	1,59	<0.001
MRI volumes							
Proportion hippocampus to ICV	0.0036 (0.00065)	15	0.0037 (0.00058)	15	*t* = 0.4	28	0.68
Proportion WMH to ICV	−2.1 (0.5)	13	−2.7 (0.7)	14	*t* = −2.5	25	0.02
Clinical scale scores							
Baseline MADRS	28.1 (8.5)	29	1.0 (1.6)	30	*t* = −17.1	57	<0.001
Baseline GDS	9.7 (3.1)	29	0.8 (1.4)	30	*t* = –14.3	57	<0.001
DSSI instrumental support	18.5 (3.0)	29	19.9 (2.3)	30	*t* = 2.0	57	0.05
DSSI social support	41.7 (5.2)	29	48.4 (6.2)	30	*t* = 4.5	57	<0.001
IADL total score	6.9 (1.7)	29	8.0 (0.2)	30	*t* = 3.6	57	<0.001
SF-36 composite	38.3 (19.9)	29	79.2 (10.7)	30	*t* = 9.9	57	<0.001
LSNS-R total	25.5 (7.6)	29	36.9 (7.3)	30	*t* = 5.9	57	<0.001
UCLA Loneliness Scale total	18.5 (7.3)	29	5.6 (4.7)	30	*t* = –8.1	57	<0.001
Activity measures							
Physical activity	0.17 (0.03)	29	0.20 (0.03)	29	*t* = 3.69	56	<0.001
Jerk	0.001 (0.0003)	29	0.001 (0.0002)	29	*t* = 4.06	56	<0.001
Entropy	2.50 (0.39)	29	2.81 (0.26)	29	*t* = 3.56	56	0.003

LLD, Late-life depression; s.d., standard deviation; df, degrees of freedom; NART, National Adult Reading Test; IQ, intelligence quotient; MMSE, Mini Mental State Examination; BMI, body mass index; MRI, magnetic resonance imaging; ICV, intracranial volume; WMH, white matter hyperintensity; MADRS, Montgomery–Åsberg Depression Rating Scale; GDS, Geriatric Depression Scale; DSSI, Duke Social Support Index; IADL, Instrumental Activities of Daily Living Scale; SF-36, 36-item Short-Form Health Survey; LSNS-R, Lubben Social Network Scale–Revised.

### Physical activity analysis

**Fig. 2.**
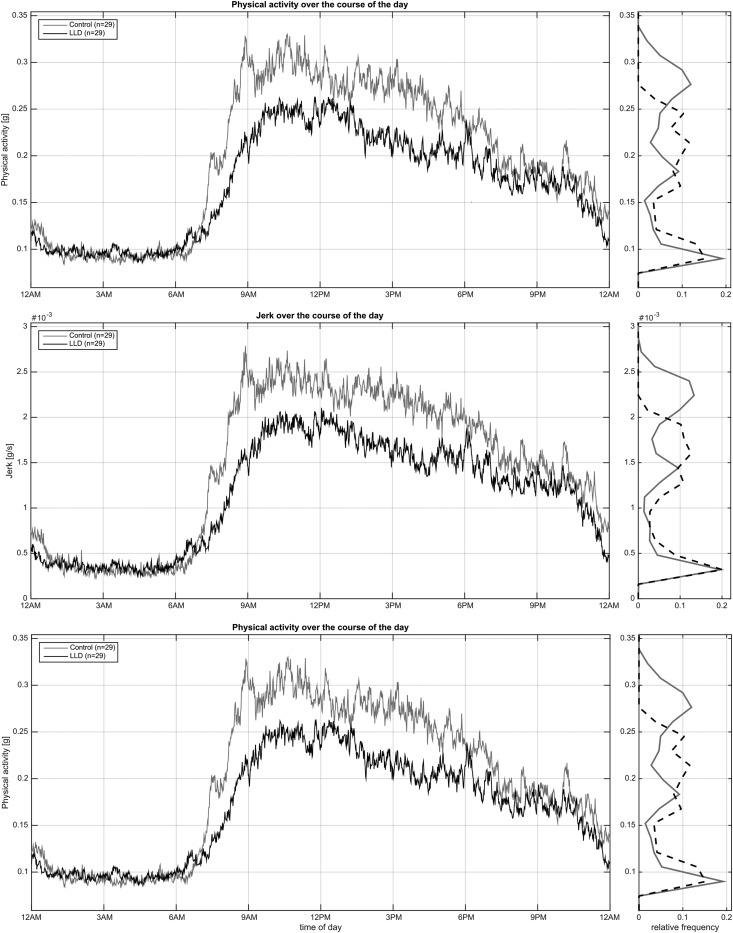
7-Day mean physical activity, jerk and entropy across a 24 h period. LLD, Late-life depression.

**Fig. 3.**
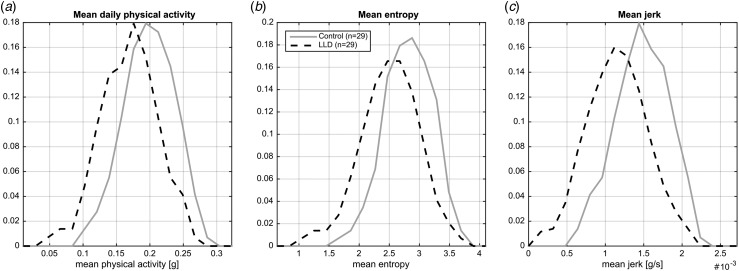
Total wear time mean activity measures. LLD, Late-life depression.

Mean 24-h activity (physical activity, jerk and entropy) levels for LLD patients and healthy controls are shown in Fig. 2. The difference in activity levels between the groups is strongest in the morning and afternoon (06.00 to 18.00 hours), while they show similar activity levels in the evening (18.00 to 00.00 hours). Fig. 3 illustrates the relative frequency (distribution) of the means over all minutes of the day for physical activity (*a*), entropy (*b*) and jerk (*c*). Two-tailed *t* tests indicate that the two groups show statistically significant differences (in mean) across all investigated signal characteristics (*p* < 0.01).

### Neuropsychological testing

There was a statistically significant group difference in neuropsychological performance when NART IQ was added as a covariate (*F*_6,52_ = 3.59, *p* < 0.005; Wilk's Λ = 0.707). Table 1 illustrates that significant between-subjects effects were observed for the *Z*-score domains of executive/working memory, attention and psychomotor speed, memory and overall performance. No significant difference was observed in short-term memory or emotional processing. For scores on individual tests contributing to these domains, see online Supplementary Table S1.

### MRI analysis

Results of group contrasts for brain and structural volumes are presented in Table 1. There were no significant differences in whole-brain volume between the LLD patients and the control participants. WMH volumes were calculated as a ratio to intracranial volume and log transformed to produce normally distributed data for analysis. Depressed patients were found to have significantly larger volumes of WMH than healthy controls. Total hippocampal volumes were also calculated as a ratio to intracranial volume: no significant differences were observed between LLD patients and controls.

### Social functioning analysis

Results of group contrasts for scores on depression scales repeated at each of the three study visits are presented in Table 1 along with scores for QoL and ADL. *t* Tests show significant group differences across all measures, with the exception of the Duke Social Support Index (DSSI) instrumental support measure, which approached significance (*t*_1,57_ = 1.98, *p* = 0.053).

### Correlation analysis

**Fig. 4.**
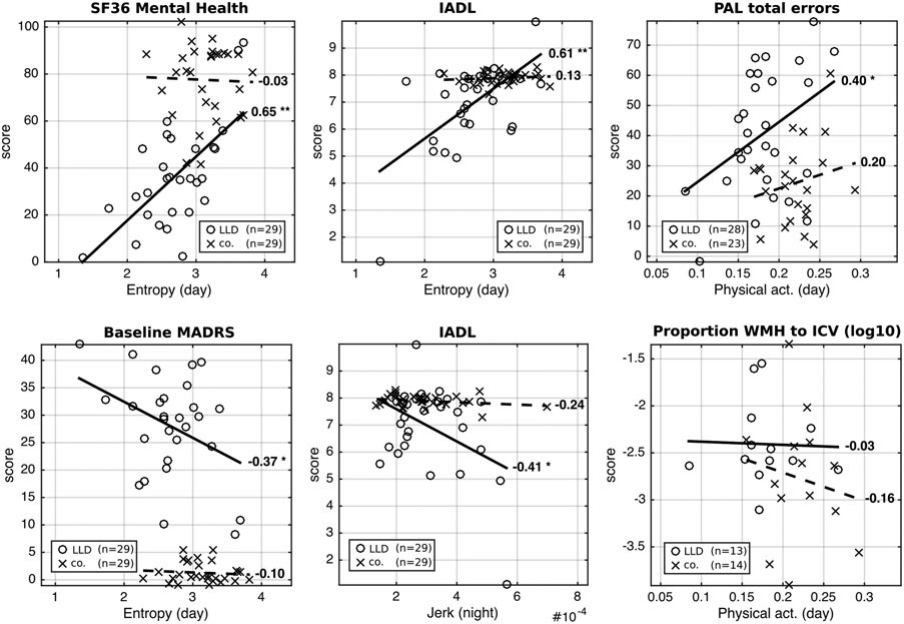
Correlation analysis between signal characteristics (mean over all days for each participant), and key study variables. *r* Values for correlation strength and direction are given per group. * *p* < 0.05, ** *p* < 0.01. Patient data are presented with a solid line of best fit, and controls with a dashed line of best fit. SF36, 36-Item Short-Form Health Survey; LLD, late-life depression; co., controls; IADL, Instrumental Activities of Daily Living Scale; PAL, paired associates learning; act., activity; MADRS, Montgomery–Åsberg Depression Rating Scale; WMH, white matter hyperintensity; ICV, intracranial volume.

Based on significant group differences, we conducted an exploratory analysis to assess the extent to which key study variables correlated with movement data. Fig. 4 illustrates partial correlations between these key variables and physical activity for depressed patients and healthy control subjects, accounting for age, BMI, pre-morbid IQ and sex. Correlations with SF-36 (Mental Health), PAL, IADL and MADRS scores were at their strongest during waking hours (06.00 to 12.00 hours: SF-36, *r* = 0.65, *p* < 0.001, *n* = 29/29; PAL, *r* = 0.40, *p* = 0.036, *n* = 28/23; IADL, *r* = 0.61, *p* < 0.001, *n* = 29/29; MADRS, *r* = −0.37, *p* < 0.05, *n* = 29/29). IADL further showed strong correlations at night time (*r* = −0.41, *p* = 0.026, *n* = 29/29). There were no significant correlations between WMH and measures of physical activity (e.g. correlation with daytime physical activity; *r* = −0.063, *p* = 0.84, *n* = 13/14).

## Discussion

The present study utilized a novel wrist-worn device to measure continuous physical movement activity objectively in older, community-dwelling adults with LLD and healthy controls over a 7-day period. This is the first study to characterize the quality of physical movement in LLD patients using an objective measure. Our results showed that patients with depression had a significant reduction in general physical activity compared with healthy controls and this difference was at its greatest during the morning and early afternoon. Assessment of specific activity parameters revealed that LLD patients showed ‘slowed’ fine motor movements (measured by ‘jerk’) compared with controls. Furthermore, these ‘slowed’ motor movements can be characterized (by the measurement of ‘entropy’) as being more repetitive and less likely to represent gesturing in LLD patients.

Broader characterization of the groups revealed generalized neuropsychological dysfunction in the LLD patients compared with controls along with increased WMH burden on MRI. IADL and QoL were also significantly reduced in LLD. Exploratory analyses demonstrated that, after accounting for age, sex and BMI differences, significant relationships were observed between selective movement parameters and aspects of episodic memory (CANTAB PAL), IADL and QoL. That the strongest relationships between key study variables and movement were found when using ‘entropy’ and ‘jerk’ indicates that future studies investigating the relationship between physical activity and LLD (or depression in general) may wish to utilize raw accelerometer output, rather than abstracted measures of physical activity.

This study had several strengths, including using a cohort of currently depressed community-dwelling adults, the use of an unobtrusive, waterproof wrist-worn activity monitor, and high levels of overall compliance for wearing the device, coupled with the long wear time (7 days).

Our study also had some limitations: we were not able to obtain MR scans for all subjects. The study was cross-sectional; hence we cannot determine causality in the relationship between physical activity and the other key variables included in our comparisons. It is important to bear in mind that some elements of the relationship between physical functioning, depression and cognitive deficits may be bi-directional and interrelated. For example, in community-dwelling, healthy older adults, increased levels of daily physical activity have been shown to be associated with reduced depressive symptomatology (Vallance *et al.* 2011; Song *et al.* 2012; Kim *et al.* 2013*b*; Loprinzi, 2013), as well as reduced cognitive decline (Yaffe *et al.* 2001). White matter integrity is also related to vascular health and there is evidence that it relates to levels of exercise (Burzynska *et al.* 2014). However, we were not able to confirm a link between fine-grained physical activity and WMH volumes in this exploratory study.

Wearable accelerometers, such as the actigraph, provide more precise assessments of locomotor activity and circadian rhythms in normal, everyday living than traditional paper-based methods (Sabia *et al.* 2014) and the utility of these devices has been recognized as a valid outcome measure for therapeutic interventions (Raoux *et al.* 1994; Winkler *et al.* 2014). The novel, wearable sensing technology we utilized in this work allowed the collection of precise data on a continuous basis. The ability to interpret direct sensor readings from a device recording continuous activity data overcomes a number of limitations of previous studies by allowing for objective assessment of movement ‘quality’ rather than arbitrarily abstracting movement data to ‘activity levels’. Previous studies in related application fields have demonstrated that more detailed movement analysis based on raw accelerometer data leads to improved outcome measures (Matthews *et al.* 2012). Accessibility of raw sensor recordings facilitates the application of sophisticated computational analysis that goes beyond gross or fine measures of movement, towards an understanding of disease-specific idiosyncrasies in physical behaviour, for example: gait analysis (Lemke *et al.* 2000), activity recognition (Bulling *et al.* 2014), automated symptom assessment (Hammerla *et al.* 2015), analysis of aggressive behaviour (Plötz *et al.* 2012), or skill assessment (Khan *et al.* 2015). Whilst these data offer huge potential as variables of interest, their utility will depend on the extent to which they are feasible and represent validated markers or surrogate markers of the disease.

Wearable technology is a rapidly developing field with implications for monitoring the long-term health and recovery of people with depression. The results of this study suggest that higher resolution analysis of accelerometer-derived physical activity may provide a suitable surrogate marker for depression in older adults. Although compliance in this study was good, further evidence is needed to assess the long-term adherence for bespoke devices. While ADL and QoL measures correlated well with physical activity, self-report measures of loneliness and social support did not, suggesting that other developing technologies may be more appropriate for investigating the link between these variables in LLD. Furthermore, since exercise has been proposed as a treatment for those with depression, including LLD (Bridle *et al.* 2012; Cooney *et al.* 2013; Mura & Carta, 2013), such devices may play a role in monitoring levels of activity when used therapeutically.

## Note

1 Open Lab: Open Movement project: https://openlab.ncl.ac.uk/things/open-movement/

## Footnotes

† The notes appear after the main text.

## Supplementary material

The supplementary material for this article can be found at http://dx.doi.org/10.1017/S0033291716002166
